# Targeting the CD47–TSP1 Axis in Abdominal Aortic Aneurysm: A Novel Immunotherapeutic Approach

**DOI:** 10.3390/ijms262211042

**Published:** 2025-11-14

**Authors:** Karolina L. Stępień, Katarzyna Janas, Stanisław Rojek

**Affiliations:** 1 Department of Molecular Biology, Faculty of Medical Sciences in Katowice, Medical University of Silesia, 40-752 Katowice, Poland; 2 Students’ Scientific Society, Department of Molecular Biology, Faculty of Medical Sciences in Katowice, Medical University of Silesia, 40-752 Katowice, Poland; s92028@365.sum.edu.pl (K.J.); s91697@365.sum.edu.pl (S.R.)

**Keywords:** abdominal aortic aneurysm, thrombospondin-1, CD47, macrophages, inflammation, extracellular matrix, immunotherapy, vascular remodeling

## Abstract

Abdominal aortic aneurysm (AAA) is a life-threatening vascular disorder characterized by progressive dilation and weakening of the abdominal aortic wall. Despite advances in surgical repair, rupture remains associated with mortality rates exceeding 65%, and no effective pharmacological therapy exists to prevent disease progression. Increasing evidence highlights chronic inflammation, extracellular matrix degradation, and immune dysregulation as central drivers of AAA pathogenesis. Among these mechanisms, the thrombospondin-1 (TSP1)–CD47 signaling axis has emerged as a critical upstream regulator of vascular inflammation. By engaging CD47, TSP1 promotes macrophage activation, impairs efferocytosis, and sustains a self-perpetuating inflammatory loop that accelerates tissue destruction. This positions the TSP1–CD47 pathway as more than a bystander in aneurysm biology, linking immune activation with structural failure of the aortic wall. The therapeutic relevance of this axis is underscored by the development of CD47-targeted agents in oncology, which restore phagocytosis and immune balance. Repurposing such strategies for vascular medicine, in combination with advanced drug delivery systems, offers a promising avenue for disease-modifying therapy in AAA. Notably, two targeted drug delivery approaches have been described: both employ bispecific targeting of CD47 in combination with a macrophage-specific marker, using immunotoxins encapsulated in liposomal carriers to enhance selectivity and therapeutic efficacy. By shifting focus from structural repair to immune modulation, targeting the TSP1–CD47 axis with these strategies has the potential to redefine the clinical management of this condition.

## 1. Introduction

Abdominal aortic aneurysm (AAA) is a chronic and potentially fatal vascular disorder defined by progressive dilation and weakening of the abdominal aortic wall. While frequently asymptomatic in its early stages, rupture remains catastrophic, with mortality rates as high as 65–85% [1,2]. Globally, AAA affects nearly 1% of adults between 30 and 79 years, with the highest burden observed among older men, especially smokers and individuals with hypertension [3,4]. Despite the implementation of screening programs and advances in surgical repair, there are currently no pharmacological therapies capable of halting aneurysm progression [5]. Consequently, surgical intervention remains the only definitive treatment. However, it is restricted to patients meeting size or growth criteria and is associated with substantial perioperative risk [6]. Current guidelines recommend elective surgical repair for asymptomatic aneurysms exceeding 5.5 cm in men or 5.0 cm in women, or for those exhibiting rapid expansion (>0.5 cm within six months) [7,8]. Below these thresholds, surveillance with regular ultrasound or CT angiography is advised. Importantly, rupture risk is influenced not only by aneurysm size but also by wall stress, inflammatory processes, and patient-specific comorbidities such as smoking, hypertension, and chronic obstructive pulmonary disease (COPD) [9,10].

In current clinical practice, population-based ultrasound screening represents the most effective strategy to reduce AAA-related mortality. Both the European Society for Vascular Surgery (ESVS, 2024) and the US Preventive Services Task Force (USPSTF, 2019) recommend one-time ultrasound screening for men aged 65–75 years with a history of smoking. Routine screening of women remains controversial, due to lower prevalence but higher rupture risk at smaller diameters [7,11,12,13]. Despite these recommendations, adherence rates vary widely across healthcare systems, and implementation remains suboptimal, particularly in low-resource settings [14]. These observations underscore the critical need for improved detection strategies and the development of novel therapeutic approaches aimed at preventing aneurysm progression and rupture.

At the molecular level, AAA pathogenesis extends beyond mechanical wall failure [15]. Chronic inflammation, extracellular matrix (ECM) degradation, oxidative stress, and vascular smooth muscle cell apoptosis collectively drive aneurysmal expansion [6,16,17]. Among these mechanisms, macrophage-mediated immune activation has emerged as a central player, fueling both inflammatory signaling and proteolytic activity [16,17]. In recent years, the thrombospondin-1 (TSP1)–CD47 signaling axis has gained particular attention as an upstream regulator of this process [18,19]. TSP1, a matricellular glycoprotein secreted by endothelial cells, smooth muscle cells, and macrophages, binds to the immunoregulatory receptor CD47 to modulate phagocytosis, nitric oxide signaling, and immune cell polarization [18]. In the aneurysmal wall, this interaction perpetuates a self-sustaining inflammatory loop by promoting M1 macrophage activation, amplifying cytokine release, and accelerating ECM degradation [18,20].

Importantly, CD47-targeted therapies are already under active investigation in oncology, where blockade of the CD47–SIRPα “don’t eat me” signal restores phagocytosis and enhances immune clearance of malignant cells [21,22]. Repurposing such strategies for vascular disease is therefore an attractive translational approach. By targeting upstream immune dysregulation rather than downstream proteolytic pathways, modulation of the TSP1–CD47 axis may provide the first disease-modifying pharmacological therapy for AAA, complementing or delaying the need for surgical repair [18,19].

The central focus of this article is the pathophysiological role of thrombospondin-1 (TSP1) and its receptor CD47 in AAA development. The TSP1–CD47 signaling axis has emerged as a critical upstream modulator of vascular inflammation. Through its effects on immune cell survival and efferocytosis, this interaction promotes sustained macrophage activation and persistent inflammation within the aneurysmal wall. Elucidating the molecular mechanisms underpinning the TSP1–CD47 axis is essential for advancing our understanding of AAA pathobiology and developing targeted therapeutic strategies. A hallmark feature of AAA pathogenesis is the presence of a self-sustaining inflammatory loop, which perpetuates tissue injury and ECM degradation. This phenomenon, and its molecular underpinnings, are discussed in detail in subsequent sections of this article.

## 2. Search Strategy and Selection Criteria

A comprehensive and structured literature search was conducted to identify studies addressing the role of CD47 signaling, particularly its interaction with TSP1 in the pathogenesis and therapeutic potential of AAA. The search covered publications from January 2000 to April 2025, focusing on recent preclinical, translational, and clinical studies (2020–2025).

Databases searched included PubMed (1 March 2025–15 September 2025), Scopus (1 March 2025–15 September 2025), Web of Science (1 March 2025–15 September 2025), Embase (1 March 2025–15 September 2025), and Google Scholar (1 March 2025–15 September 2025), supplemented by ClinicalTrials.gov (1 March 2025–15 September 2025) and pharmaceutical development databases to identify ongoing or completed anti-CD47 trials (e.g., magrolimab, lemzoparlimab). Reference lists of key studies and reviews were also screened. Search terms combined MeSH and free-text keywords across four thematic clusters: disease focus (“abdominal aortic aneurysm”, “vascular inflammation”); molecular targets (“CD47”, “thrombospondin-1”, “TSP1”); therapeutic relevance (“anti-CD47 antibody”, “efferocytosis”, “magrolimab”); and mechanistic pathways (“matrix metalloproteinases”, “macrophage polarization”). Boolean operators and field filters were applied to refine results.

Eligible studies included preclinical or clinical investigations of CD47/TSP1 in AAA or related vascular diseases, mechanistic analyses of macrophage-driven inflammation and matrix degradation, and reports on CD47-targeted therapeutics relevant to vascular pathology. Exclusion criteria comprised non-English publications, purely oncologic studies without vascular relevance, and non-peer-reviewed materials.

## 3. Molecular Basis of AAA Development

The pathogenesis of AAA is a multifactorial process driven by chronic inflammation and progressive structural weakening of the vascular wall. Its development results from an interplay between genetic predisposition and environmental factors that trigger pathological alterations in aortic tissue [23]. Major environmental contributors include cigarette smoking, advanced age, male sex, and hypertension [24]. Among these, smoking is the strongest risk factor, as it induces oxidative stress, activates proteolytic enzymes, and promotes inflammatory cell infiltration into the aortic wall [25,26]. Variants in genes encoding extracellular matrix–degrading enzymes, such as matrix metalloproteinases (MMP-2, MMP-9), as well as polymorphisms in genes regulating inflammatory cytokines, including interleukin-6 (IL-6) and tumor necrosis factor-alpha (TNF-α), have been associated with increased AAA susceptibility [27,28]. Familial aggregation studies further support a hereditary component, with first-degree relatives of affected individuals showing a significantly elevated risk [29]. In addition, epigenetic modifications, such as DNA methylation and histone remodeling, are gaining attention as potential regulators of gene expression in AAA, influencing vascular remodeling and inflammatory responses even in the absence of conventional risk factors [30].

Clinically, these molecular alterations correspond to measurable aortic expansion during follow-up imaging. Data from large prospective cohorts, including the UK Small Aneurysm Trial, indicate an average annual growth rate of 2–3 mm, with smokers and hypertensive patients exhibiting faster progression [10,31]. Conversely, diabetes mellitus appears paradoxically protective, possibly due to increased extracellular matrix cross-linking and reduced proteolytic activity [32]. These findings highlight the need for individualized clinical surveillance.

Histopathologically, AAA is marked by persistent vascular inflammation, apoptosis of vascular smooth muscle cells (VSMCs), oxidative stress, and progressive degradation of the ECM [15,33]. MMPs, particularly MMP-2 and MMP-9, are central mediators of ECM breakdown. These enzymes degrade key structural proteins, including elastin and collagen, thereby compromising the mechanical stability of the aortic wall. Elevated levels of these enzymes are consistently detected in aneurysmal tissue and are mainly secreted by infiltrating macrophages and neutrophils. An imbalance between MMPs and their endogenous inhibitors, tissue inhibitors of metalloproteinases (TIMPs), further accelerates ECM degradation. Consequently, MMPs are considered promising therapeutic targets for attenuating AAA progression [34,35]. Beyond proteolytic enzymes, growing attention has shifted toward matricellular proteins that actively regulate vascular inflammation and remodeling. Among them, thrombospondin-1 (TSP1) has emerged as a particularly important mediator, as its signaling through CD47 amplifies chronic immune activation and ECM degradation. These properties position TSP1 as a key contributor to AAA pathogenesis and provide a rationale for exploring its role in greater detail.

### 3.1. TSP-1 Role in AAA Progression

Thrombospondins (TSPs) are a multifunctional family of matricellular proteins that regulate ECM dynamics and influence key biological processes such as tissue remodeling, inflammation, and vascular homeostasis [36,37,38]. This family consist of five members (TSP1–TSP5), secreted by various cell types including platelets, endothelial cells, macrophages, smooth muscle cells, and fibroblasts. Under physiological conditions, TSPs modulate cell–matrix interactions, inhibit angiogenesis, promote platelet aggregation, and regulate immune responses, thereby contributing to tissue repair, hemostasis, and immune homeostasis [36,37,38]. TSPs are also implicated in numerous pathological processes, including cancer, cardiovascular diseases (CVDs), autoimmune disorders, diabetes, and metabolic syndromes [36,37,38,39]. Among them, TSP1 has received particular attention due to its interaction with CD47, a key signaling axis involved in immune evasion, inflammation, and vascular dysfunction [20,36,37]. The TSP1–CD47 pathway plays a central role in the pathogenesis of AAA [20,40,41], atherosclerosis [38], myocardial infarct ion [42], and hypertension [38,43]. While other thrombospondins may also contribute to disease mechanisms [38,43,44], TSP1 remains the most extensively studied [36,37,38]. Recent work by Heng Pan 2024 highlighted the pleiotropic effects of TSP1 in CVDs and emphasized the need for further investigation [36]. Elevated TSP1 levels have been associated with increased vascular inflammation and disease progression, including in AAA [20,40,41], acute coronary syndrome [45], chronic kidney disease [46], hemodialysis patients with cardiovascular disease [47], and diabetic vascular complications [39]. However, some studies report inconsistent findings [36,37,38], underscoring the complexity and context-dependence of TSP1 activity. Nevertheless, accumulating evidence supports elevated TSP1 expression as a common feature of cardiovascular pathology, particularly in AAA [20,40,41]. This highlights the translational potential of TSP1-mediated signaling as both a biomarker and a therapeutic target in vascular disease [20,36,37,38].

Functionally, TSP1 exhibits antiangiogenic, pro-inflammatory, adhesive, regulatory, and fibrotic properties [36,37,38]. During chronic inflammation, its regulation may become disrupted, driving pathological tissue remodeling [20,36,37,38]. A comprehensive understanding of these effects requires insight into the protein family’s structural organization.

**Figure 1:**
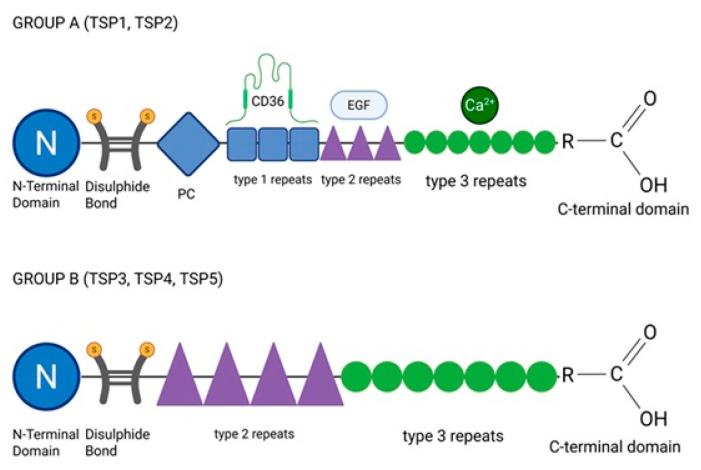
Classification of thrombospondins based on their structure and served function. TSPs are classified into two structural and functional groups based on domain composition and oligomerization. Group A (TSP1, TSP2) forms trimers and contains a procollagen homology domain (PC), type 1 repeats (TSRs), EGF-like type 2 repeats, and calcium-binding type 3 repeats. TSRs mediate anti-angiogenesis, ECM remodeling, and pro-inflammatory signaling, mainly via CD36 and transforming growth factor (TGF-β) activation. Group B (TSP3, TSP4, TSP5/COMP) forms pentamers and lacks TSRs and PC domain. Instead, it features extended type 2 and type 3 repeat involved in tissue development, angiogenesis, and fibrosis regulation. Despite lacking TSRs, Group B TSPs retain context-dependent regulatory in cardiovascular and connective tissue homeostasis, with both pro- and anti-inflammatory effects. Created in BioRender. Janas, K. (2025) https://BioRender.com/a0lqknm (accessed on 3 November 2025).

Thrombospondins are structurally and functionally divided into two distinct groups, A and B (Figure 1) [36,37,38]. Group A includes TSP1 and TSP2, which form trimeric complexes stabilized by disulfide bonds [36,37]. Each monomer contains an N-terminal domain, a procollagen homology domain (PC), and multiple repeat motifs (types I–III). Type I (thrombospondin type 1 repeats, TSRs), type II (EGF-like repeats, epidermal growth factor-like domains), and type III (calcium-binding repeats) mediate receptor interactions [36,37]. TSP1 terminates with a C-terminal globular domain that binds ligands such as CD36, while its N-terminal domain mediates adhesion via glycosaminoglycans, calreticulin, and integrins [36,37]. A key structural feature of both TSP1 and TSP2 is the type 1 repeat (TSR) within their polypeptide chains [36,37]. Their trimeric structure is stabilized by disulfide bonds in the coiled-coil region (Figure 1) [36,37]. Group A thrombospondins are implicated in various pathological processes, including apoptosis, pulmonary hypertension, atherosclerosis, fibrosis, and vascular inflammation [33,34,35,38,46]. Group B includes TSP3, TSP4, and TSP5, also known as cartilage oligomeric matrix protein (COMP). These molecules are extracellular matrix glycoproteins characterized by a pentameric structure, distinguishing them from the trimeric group A TSPs. Each monomer contains a conserved N-terminal coiled-coil region, which enables pentamer formation via disulfide bonds, EGF-like type II repeats, calcium-binding type III repeats, and a C-terminal globular domain [36,44]. Unlike group A members, group B TSPs lack type I repeats and procollagen-like domains. Type III repeats ensure structural integrity and mediate matrix interactions, while the C-terminal domain supports ligand binding and functional specificity. These structural features underlie their unique roles in tissue development and homeostasis. Group B thrombospondins contribute to cardiomyocyte integrity and modulate key pathophysiological processes, such as inflammation, angiogenesis, and fibrosis [28,36,48]. Group B thrombospondins contribute to cardiomyocyte integrity and modulate key pathophysiological processes, such as inflammation, angiogenesis, and fibrosis. They also help mitigate hypertension and vascular calcification and can exert anti-inflammatory effects, emphasizing their multifaceted, context-dependent functions in cardiovascular health and disease [36,49].

The presence of TSRs in group A thrombospondins constitutes the main structural and functional distinction between the groups. TSRs, first identified during molecular cloning of TSP1 by Lawler and Hynes, are short conserved motifs of ~60 amino acids, enriched in tryptophan and arginine, forming a three-layered fold stabilized by disulfide bonds. These domains mediate interactions with glycosaminoglycans and receptors such as CD36, exert anti-angiogenic effects via caspase-3 activation, regulate MMP activity, and activate latent TGF-β [45]. The positively charged front face binds to negatively charged ligands, such as heparan sulfate proteoglycans. In TSP1, TSRs exert anti-angiogenic effects via CD36-mediated activation of caspase-3, inducing endothelial cell apoptosis. They also regulate MMPs, activate latent TGF-β through conserved motifs (e.g., the arginine–phenylalanine–lysine sequence that enables TGF-β activation, RFK), and facilitate interactions with ECM components like collagen, fibronectin, and von Willebrand factor. Beyond TSPs, TSR are found in proteins such as ADAMTS1 (a disintegrin and metalloproteinase with thrombospondin motifs 1), SCO-spondin (subcommissural organ spondin), and R-spondins (roof plate–specific spondins), contributing to ECM anchoring, axonal guidance, and Wnt signaling [36,50,51,52].

The involvement of TSPs in CVD pathogenesis is well documented and supported by multiple studies [45,53,54]. A central mechanism in which TSPs, particularly TSP1, play a pivotal role is the initiation and propagation of inflammation, leading to degradation of connective tissue within the circulatory system, especially in the vascular walls. TSP1 is primarily released from damaged tissue in response to external factors such as cigarette smoke, which promotes oxidative stress, protease activation, and inflammation. TSP1 interacts with immune cells migrating to inflammatory sites. A key event is the binding of TSP1 to CD47 on macrophages, triggering M1 polarization, monocyte recruitment, cytokine release, and immune cell infiltration. This interaction activates macrophages and induces autocrine TSP1 secretion, amplifying the pro-inflammatory response. This creates a self-sustaining molecular circuit termed the inflammatory loop, discussed further in this study. Disruption of this loop may underlie chronic inflammatory conditions such as AAA. Understanding this mechanism is critical for elucidating AAA pathogenesis and may support the development of targeted therapies aimed at modulating this pathway in AAA and other inflammatory CVDs. Beyond inflammation, TSPs are implicated in other pathological processes in CVDs. A study published in JACC: Basic to Translational Science demonstrated that uremic toxins such as indoxyl sulfate upregulate TSP1 in both animal models and human cardiomyocytes, contributing to cardiac remodeling in chronic kidney disease (CKD). Elevated TSP1 levels were associated with myocardial hypertrophy, fibrosis, and diastolic dysfunction, whereas its inhibition yielded cardioprotective effects, suggesting potential therapeutic applications [53]. Additionally, a study in Respiratory Research identified TSP4 as contributing to pulmonary vascular remodeling in patients with congenital heart defects and secondary pulmonary hypertension. TSP4 promoted smooth muscle cell proliferation and structural changes in the vessel wall, potentially accelerating disease progression [46]. A clinical study from Beijing further associated elevated plasma TSP1 levels with worse outcomes in acute coronary syndrome (ACS), including higher mortality, recurrent ischemia, and in-hospital heart failure, highlighting its potential as a prognostic biomarker [45].

### 3.2. CD47 Receptor: Structure and Role in Immune Regulation

**Figure 2:**
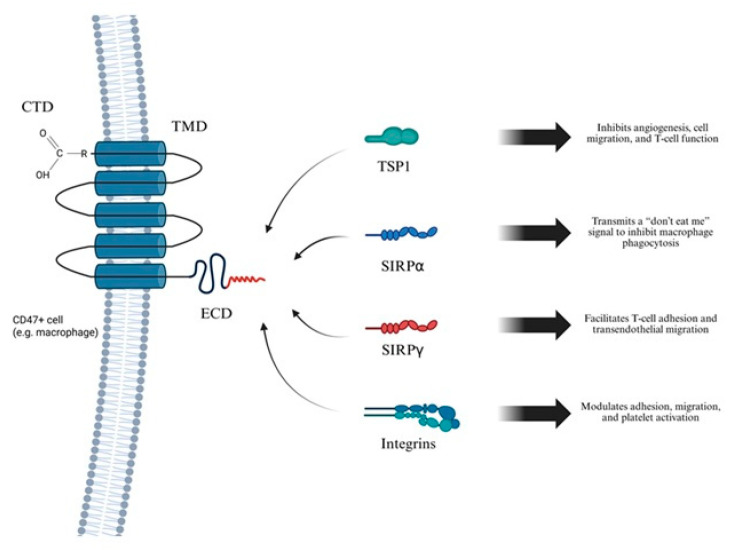
Overview of CD47 structure and function. This figure depicts the molecular architecture of the CD47 receptor and its interactions with key extracellular ligands that regulate a range of cellular processes, including immune responses, vascular homeostasis, and inflammation. CD47 is a five-transmembrane domain (5-TM) receptor consisting of an extracellular immunoglobulin variable-like domain (IgV), TMD, and CTD. CD47 is broadly expressed on immune and endothelial cells, including macrophages. Created in BioRender. Janas, K. (2025) https://BioRender.com/65zr6no (accessed on 3 November 2025).

CD47, also known as integrin-associated protein (IAP), is a ubiquitously expressed transmembrane glycoprotein involved in numerous physiological and pathological processes. It consists of an N-terminal immunoglobulin variable (IgV)-like extracellular domain, five transmembrane helices, and a short cytoplasmic tail, with multiple isoforms arising from alternative splicing of its cytoplasmic region. CD47 is expressed on a wide range of cells, including erythrocytes, platelets, endothelial cells, and immune cells. Functionally, CD47 is a key regulator of immune homeostasis. Through interaction with signal regulatory protein alpha (SIRPα) on phagocytic cells, it transmits a “don’t eat me” signal that inhibits phagocytosis of self-cells. It also modulates cell adhesion, migration, and apoptosis via interactions with integrins and TSP1. The CD47-TSP1 axis further contributes to nitric oxide signaling inhibition, influencing vascular tone and angiogenesis. Overexpression of CD47 in various cancers promotes immune evasion and highlights its potential as therapeutic target. CD47 is particularly abundant on macrophages, facilitating diverse ligand-mediated signaling events under both physiological and pathological conditions. Detailed molecular characterization of CD47 is crucial to understanding the pathogenesis of several diseases, including AAA. Its structural features include an extracellular domain (ECD) with N-terminal immunoglobin variable (IgV)-like domain that binds to SIRPα (signal regulatory protein), delivering an anti-phagocytic signal; a transmembrane domain (TMD) composed of five transmembrane helices that stabilize ECD on the membrane; and a cytoplasmic domain (CTD), which is short and alternatively spliced, producing four isoforms with tissue-specific functions (Figure 2) [55].

**Figure 3:**
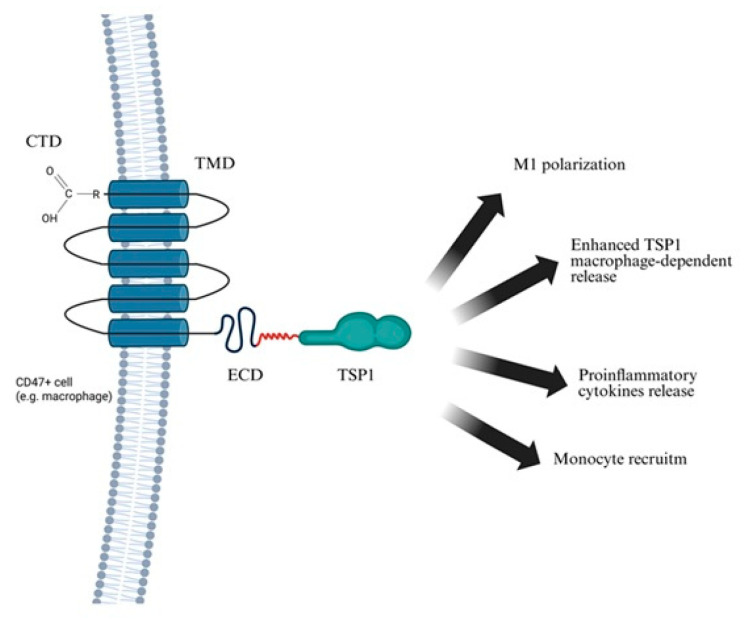
Consequences of TSP1–CD47 ligation. The figure illustrates the interaction between TSP1 and CD47 on the surface of a CD47-positive cell (e.g., macrophage). TSP1 binds to the extracellular domain of CD47, triggering downstream signaling pathways that modulate immune activation and inflammation. This interaction plays a key role in both physiological regulation and pathological processes, including chronic inflammation and cardiovascular disease. Created in BioRender. Janas, K. (2025) https://BioRender.com/ppslhvq (accessed on 2 November 2025).

Due to the pivotal role of CD47 in regulating immune responses and cellular signaling, a thorough understanding of its ligands and their interaction mechanisms is essential (Figure 3). Most ligands bind to the extracellular domain of CD47. The principal agonists of this receptor include SIRPα, SIRPγ, TSP1 and integrins. SIRPα is predominantly expressed on myeloid cells, including macrophages and dendritic cells. It binds to the IgV domain of CD47, leading to phosphorylation of immunoreceptor tyrosine-based inhibitory motifs (ITIMs) and recruitment of Src homology 2 (SH2) domain-containing phosphatases SHP-1 and SHP-2, which mediate an inhibitory “don’t eat me” signal that blocks phagocytosis. Many cancer cells overexpress CD47 to avoid immune clearance, making the CD47–SIRPα axis a promising target in cancer immunotherapy [56]. SIRPγ is predominantly expressed on T lymphocytes and binds with CD47 with lower affinity than SIRPα. This interaction promotes T cell adhesion to endothelial cells and facilitates their transendothelial migration. Unlike SIRPα, SIRPγ possesses a short cytoplasmic tail lacking ITIM motifs, indicating a potential role as a co-receptor or involvement in unidirectional signaling [56]. TSP1, is a secreted glycoprotein involved in angiogenesis, immune regulation, and cellular metabolism. It binds CD47 with high affinity, modulating key processes such as cell migration, adhesion, proliferation, and apoptosis. The TSP1/CD47 interaction suppresses T cell function by limiting TCR-dependent signaling and activation. This pathway also contributes to metabolic diseases, such as diabetes, by disrupting mitochondrial homeostasis and cellular energy balance. Dysregulation of the TSP1/CD47 axis promotes tumor progression through immune evasion and inhibits antitumor T cell responses. Moreover, enhanced TSP1/CD47 signaling is associated with impaired tissue repair and chronic inflammation in cardiovascular and metabolic disorders [57,58]. Integrins are transmembrane receptors that mediate cell adhesion, migration, and signal transduction. CD47 is associated with integrins such as αvβ3 and α2β1, modulating their activation and promoting cell spreading and motility. These interactions affect critical biological processes, including immune cell trafficking, platelet aggregation, and wound healing. The CD47–integrin complex also contributes to the regulation of extracellular matrix response and supports cell survival under stress conditions [59].

CD47 plays a key role in several pathological conditions by regulating immune evasion, inflammation, and cell survival. In cancer, many tumor cells overexpress CD47 to avoid phagocytosis, and therapeutic blockade of the CD47–SIRPα axis is being explored to enhance macrophage-mediated clearance [20,60]. Another CD47 ligand, TSP 1, further promotes immune evasion and metabolic dysfunction by inhibiting T cell activation and disrupting mitochondrial homeostasis. In neurodegenerative diseases, such as multiple sclerosis, CD47 deficiency has been associated with reduced immune activation and milder disease progression. In atherosclerosis, elevated CD47 levels hinder the clearance of apoptotic cells, promoting chronic inflammation and contributing to plaque instability [20,61,62]. CD47 also modulates integrin activity, affecting cell adhesion and migration, which influences leukocyte recruitment and vascular remodeling in inflammatory settings. At the molecular level, CD47 (Cluster of Differentiation 47) expression is regulated by transcription factors such as MYC (myelocytomatosis oncogene) and NF-κB (nuclear factor kappa-light-chain-enhancer of activated B cells). Its ligation can trigger caspase-independent cell death in malignant cells [63,64]. Moreover, CD47 is implicated in connective degradation, often observed in CVDs, where it enhances inflammation by promoting phagocytosis, leukocyte infiltration and secretion of pro-inflammatory cytokines [20,65]. Targeting CD47 has emerged as a promising therapeutic approach, particularly in cancer and CVDs. Strategies such as monoclonal antibodies, SIRPα-Fc fusion proteins, and small molecule inhibitors, have demonstrated potential in restoring immune clearance and dampening inflammation. Clinical trials are ongoing to evaluate safety and efficacy of these therapies, especially in combination with other immunomodulatory agents. Moving forward, the development of more selective CD47-targeted treatments with reduced off-target effects may improve therapeutic outcomes and expand their clinical utility. In this context, the rationale for pursuing refined CD47-directed therapies becomes clearer when contrasted with the lack of clinical efficacy observed for other targeted approaches, most notably MMP inhibitors.

## 4. Lack of Clinical Efficacy of MMP Inhibitors in the Management of AAA

Given the role of MMPs in AAA pathogenesis, they are considered promising therapeutic targets [17]. The catalytic domain of MMPs contains a zinc ion coordinated by histidine residues, forming the active site essential for enzymatic activity [66,67,68]. MMP inhibitors mimic TIMPs by binding to the zinc-binding domain of MMPs, thereby blocking catalytic activity and preventing proteolytic cleavage of protein substrates [66]. Endogenous TIMPs, present in the ECM, reversibly regulate MMP activity in vivo through the same mechanism [67]. They suppress both pathological overexpression and physiological levels of MMPs, which represents a significant limitation to their use as therapeutic agents due to the risk of disrupting normal tissue homeostasis [69]. In the context of AAA the protective role of TIMPs in aneurysm progression was demonstrated. Timp1 gene knockout in induced AAA mice models consequences in aortic diameter increase and greater elastin loss [70]. Initial strategies focused on chelating the zinc ion within the zinc-activation site on MMPs [71].

### 4.1. Therapeutic Potential of MMP Inhibition in AAA

Broad-spectrum MMP inhibitors have been evaluated in the context of AAA. The non-selective inhibitor doxycycline initially showed promise in animal models and early clinical trials, demonstrating reduced MMP-9 levels and slower aneurysm growth [70]. However, larger and long-term studies yielded inconsistent or negative results [70], likely due to doxycycline’s broad, non-specific mechanism that fails to address the complex pathophysiology of AAA. This ultimately led to the discontinuation of its clinical development for AAA [70,72]. Notably, even genetic knockout of MMP-12 in animal models also failed to prevent AAA progression, highlighting the redundancy of proteolytic and inflammatory pathways [73]. Furthermore, the impact of MMP-12 deletion remains inconsistent, across studies, further complicating the development of selective MMP-targeted therapies [73]. Importantly, inhibition of MMPs alone, even those primarily involved in ECM degradation, has proven insufficient to halt the broader pathogenic cascade driving AAA. Beyond MMP inhibition, several pharmacological approaches have been clinically tested. Statins have shown modest benefits in slowing aneurysm growth, potentially due to pleiotropic anti-inflammatory and endothelial-stabilizing effects [74]. ACE inhibitors and angiotensin receptor blockers have yielded inconsistent results, whereas β-blockers failed to demonstrate significant benefit in randomized trials [75,76]. These findings underscore the persistent lack of effective pharmacotherapy and the urgent need for novel immunomodulatory strategies such as CD47 blockade.

### 4.2. Translational Applications and Broader Therapeutic Challenges

Matrix metalloproteinase inhibitors have been investigated in various conditions involving dysregulated ECM remodeling and tissue invasion, such as solid tumors (e.g., breast, pancreatic, ovarian), osteoarthritis, post-myocardial infarction remodeling, pulmonary fibrosis, multiple sclerosis, and periodontal disease [67,77]. Although cipemast, an inhibitor of MMP-1, -3, and -9, was evaluated in osteoarthritis and rheumatoid arthritis, it failed to prevent joint degeneration [67]. A significant limitation of broad-spectrum MMP inhibitors has been their adverse safety profile including musculoskeletal toxicity (arthralgia, myalgia, stiffness) and gastrointestinal disorders. Many anti-arthritic MMP inhibitors were withdrawn due to musculoskeletal syndrome (MSS) [71]. Furthermore, blocking MMPs may trigger compensatory mechanisms through altered gene expression, diminishing therapeutic efficacy [78]. Despite promising preclinical data, clinical results have remained inconsistent, though ongoing research is justified by their therapeutic potential [71]. Batimastat was the first broad-spectrum MMP inhibitor to be tested in patients [78]. As a hydroxamic acid, it binds zinc ions in MMP active site, preventing substrate cleavage [69,78]. Although early trials in solid tumors showed partial responses, clinical development was halted due to poor bioavailability and adverse effects [69]. In 2024, renewed interest in batimastat emerged for hematologic malignancies, particularly acute myeloid leukemia (AML), which showed the highest in vitro sensitivity among tested cell lines (AML, MDS, MM) [78]. However, all clinical trials to date have been limited to solid tumors, leaving hematologic applications underexplored [78]. Batimastat was discontinued due to solubility issues and toxicities such as peritonitis and liver dysfunction [79]. Its successor, marimastat, showed improved bioavailability but was also withdrawn due to fibrosis and toxicity [79]. Other broad-spectrum inhibitors, including prinomastat and tanomastat, failed in Phase III trials due to side effects (arthritis, bone marrow suppression, gastrointestinal toxicity, thromboembolism) and their limited impact on tumor size [79]. These outcomes alone have shifted focus toward developing selective MMP inhibitors. Targeting noncatalytic domains, which differ among MMP isoforms, offers enhanced specificity and fewer off-target effects [79].

As of 2025, nanoparticle-based delivery systems are being explored to improve MMP inhibitor efficacy, particularly in glioblastoma, where MMP-2 and MMP-9 drive ECM degradation and tumor invasion. Nanostructured lipid carriers (NLCs) have been developed for targeted batimastat delivery, aiming to improve stability, tumor specificity, and reduce systemic toxicity [80]. This strategy may also benefit conditions like AAA [69]. Beyond such delivery-based approaches, attention must also turn to the primary biological contributors to vascular degeneration, among which macrophages represent a key focus.

## 5. Macrophage-Mediated Remodeling and Destruction of the Aortic Wall

Given the role of macrophages as a major source of MMP-9 and MMP-2, and pro-inflammatory cytokines in AAA [16,17,81,82,83,84], we aimed to further investigate their involvement, with a particular focus on activation dynamics and contribution to the inflammatory milieu. Moreover, monocytes and macrophages represent the predominant immune cell population infiltrating the aortic wall during early stage of AAA, highlighting their potential not only as therapeutic targets but also as key modulators in strategies aimed at preventing AAA progression [84].

### 5.1. Monocyte Recruitment and Differentiation

Macrophages present in AAA lesions are primarily derived from bone marrow–originating monocytes circulating in the blood [85,86]. These monocytes are recruited to inflamed tissues in response to chemokine signaling, with C-C motif ligand 2 (CCL2, also known as MCP-1) playing a key role [87,88,89]. CCL2 binds to CCR2 (C-C chemokine receptor type 2), a receptor highly expressed on Ly6C^high monocytes in mice and CD14^+^ CD16^−^ monocytes in humans, subsets with potent pro-inflammatory potential [90,91,92]. These monocytes accumulate in the aortic wall during AAA development in murine models, contributing to inflammation and tissue remodeling [90,93].

Once infiltrated, monocytes differentiate into macrophages and polarize under local inflammatory cues. M1 polarization, marked by CD86 expression, leads to the secretion of pro-inflammatory cytokines and predominates in AAA tissue, driving chronic inflammation, ECM degradation, and aneurysm progression [84,93]. A balanced M1/M2 macrophage ratio has been linked to aneurysm stability, whereas an M1-dominant profile correlates with increased rupture risk [94]. M1 macrophages also localize to sites of vascular injury, where they further amplify local inflammation [94].

### 5.2. Activation of Macrophages in AAA

Within the aortic wall, macrophages are activated by various pro-inflammatory stimuli that drive polarization toward the M1 phenotype [95]. Key inducers include cytokines such as TNF-α and interleukin-1 beta (IL-1β), which not only activate macrophage but also promote further secretion of inflammatory mediators, including TNF-α itself [96]. This establishes a feed-forward inflammatory loop that amplifies local inflammation and increases MMP-9 production [97,98]. TSP1 also enhances macrophage activation. Additionally, damage-associated molecular patterns (DAMPs) released by stressed or necrotic VSMCs and ECs (endothelial cell), particularly under oxidative stress, act as potent sterile inflammatory signals that sustain macrophage activation and the inflammatory environment.

### 5.3. Macrophage-Mediated Structural Remodeling of the Aortic Wall

**Figure 4:**
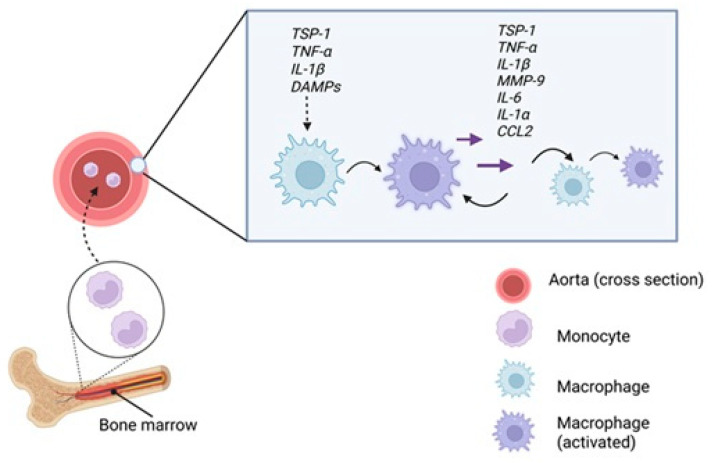
M1 polarization within the aortic wall. Monocytes are recruited from the bone marrow into the bloodstream and migrate to the inflamed aortic wall. There, exposure to pro-inflammatory stimuli such as TSP1, TNF-α, IL-1β, DAMPs released from stressed or necrotic VSMC and EDs, promotes their polarization into M1 macrophages. These, in turn, secrete inflammatory mediators including TSP1, TNF-α, IL-1β, IL-6, IL-1α, CCL2 and MMP-9, amplifying local inflammation and reinforcing M1 polarization in neighboring macrophages through autocrine and paracrine signaling, thus sustaining the pro-inflammatory state of the aortic wall. Created in BioRender. Janas, K. (2025) https://BioRender.com/1iegpxp (accessed on 2 November 2025).

Activated M1 macrophages play a central role in ECM remodeling and vascular degeneration in AAA by secreting MMP-9 and MMP-2, pro-inflammatory cytokines (IL-1α and IL-6), CCL 2, and TSP1 (Figure 4) [20,94]. Inflammatory macrophages are the primary source of elevated TSP1 in AAA [20], and both histological analyses and murine models confirm macrophages as the predominant producers of MMP-9 in aneurysmal tissue [16,97]. Beyond serving as a marker of macrophage activation, TSP1 modulates the immune environment by activating latent TGF-β, promoting leukocyte adhesion, and inhibiting angiogenesis, factors that collectively contribute to vascular wall degeneration. These mediators not only contribute to ECM degradation but also sustain chronic vascular inflammation. Notably, IL-1α constitutively expressed in endothelial and other cells, is markedly upregulated by oxidative stress and cytokines such as IL-1β and IL-1α itself [99]. Further amplifying inflammation, NF-κB signaling, activated by IL-1β and TNF-α, induces transcription of pro-inflammatory genes, including TNF-α [100].

### 5.4. Interferon-Inducible Macrophages in AAA Pathogenesis

Sheng et al. identified a distinct subset of interferon-inducible monocytes/macrophages linked to AAA progression and rupture risk in murine models [89]. Transcriptomic analysis revealed that activation of the cyclic GMP-AMP synthase–stimulator of interferon genes (cGAS–STING) and Janus kinase–signal transducer and activator of transcription (JAK–STAT) pathways induced type I interferon production, driving the differentiation of monocytes/macrophages into interferon-inducible cells (IFNICs) [89]. Unlike classical M1/M2 subsets, IFNICs are characterized by heightened sensitivity to type I interferons and a unique transcriptional profile, including expression of interferon-stimulated genes (ISGs) such as Isg15 and Ifit1 [89,101]. Myeloid-specific deletion of Sting1 or Ifnar1 in murine models significantly reduced AAA incidence, rupture rate, and aortic diameter, highlighting the pathogenic role of these macrophages in AAA development [89].

### 5.5. Therapeutic Potential of M2 Macrophages

In the study by Shinichi Ashida et al., administration of M2 macrophages in murine AAA models significantly reduced the expression of IL-1β, IL-6, and CCL2, and limited elastin degradation compared to saline-treated controls [94]. The injected M2 macrophages migrated to the aortic wall without transitioning into the M1 phenotype [94]. Co-culture experiments further showed that M2 macrophages downregulated MMP-2 and MMP-9 expression in M1 macrophages, while upregulating anti-inflammatory cytokines IL-10 and TGF-β under a 1:1 M1/M2 ratio [94]. This suggests that paracrine modulation by M2 macrophages is context-dependent and influenced by the local microenvironment. Notably, M2 macrophages secrete pro-fibrotic mediators, including TGF-β and VEGF. Transcriptomic studies of systemic sclerosis (SSc) skin biopsies consistently identify M2 activation as a hallmark of the disease, underscoring a therapeutic dilemma: while M2 macrophages have anti-inflammatory potential, their pro-fibrotic activity may contribute to maladaptive tissue remodeling in fibrotic conditions like SSc [102].

## 6. Inflammatory Loop of AAA

**Figure 5:**
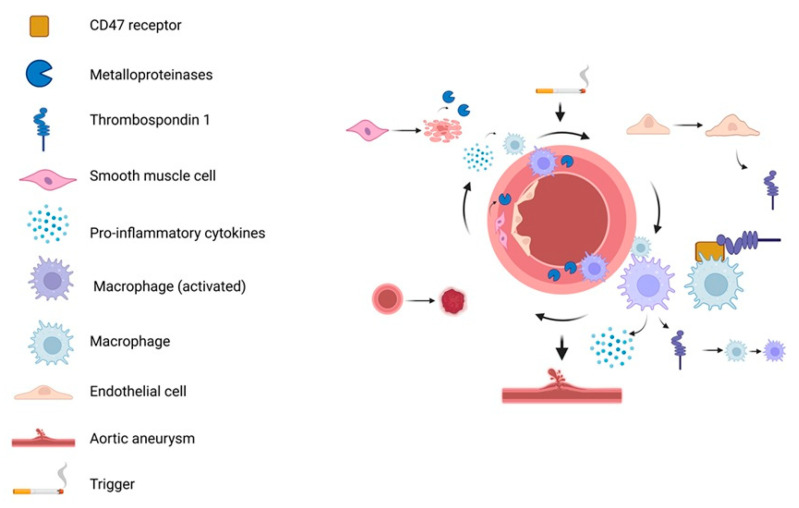
Inflammatory loop in AAA. Initial triggers induce ECs injury and release of TSP1 which binds to CD47 on macrophages, promoting their pro-inflammatory polarization. Activated macrophages infiltrate the aortic wall, secreting cytokines and MPs that degrade the ECM and induces death. SMCs can also release MMPs, further exacerbating tissue damage and inflammation. This feedback loop sustains macrophage activation and cytokine release, ultimately leading to aneurysm formation. Created in BioRender. Janas, K. (2025) https://BioRender.com/o0c1u0i (accessed on 30 October 2025).

To mechanistically link risk factors with aneurysm formation, a stepwise model has been proposed, emphasizing TSP1–CD47–mediated macrophage activation and the resulting inflammatory loop (Figure 5). Following vascular injury, TSP1 is released by ECs, SMCs, and platelets [21,100,103]. TSP1 binds to CD47 on macrophages, promoting their polarization into the M1 pro-inflammatory phenotype [36]. These macrophages infiltrate the aortic wall and release MMPs, especially MMP-9, driving ECM degradation. SMCs also contribute to MMP-2 and MMP-9 production [69,94]. Importantly, TSP1-CD47 interaction is critical for macrophage entry into the ECM [20]. ECM breakdown and SMC apoptosis stimulate cytokine release (e.g., IL-6, IL-1β, TNF-α, iNOS), creating a chemotactic gradient that recruits additional monocytes [82,94]. This forms a self-sustaining inflammatory loop, wherein ongoing tissue damage perpetuates TSP1 release and CD47-mediated macrophage activation [36]. Progressive loss or phenotypic switching of vascular SMCs has been observed in murine AAA models, reinforcing their role in aneurysm formation and wall weakening [104,105].

## 7. Anti-CD47 mo-Ab in Cancer

The CD47 receptor has emerged as a clinically relevant immunotherapeutic target in oncology due to its key role in tumor immune evasion. By binding to signal regulatory protein alpha (SIRPα) on macrophages and dendritic cells, CD47 transmits a “don’t eat me” signal that inhibits phagocytosis and allows malignant cells to escape immune surveillance [21]. Blocking this interaction with monoclonal antibodies restores the capacity of innate immune cells to eliminate tumor cells, making CD47 an attractive target, particularly in hematologic malignancies [22]. Monoclonal antibodies such as m agrolimab, lemzoparlimab, and bispecific antibody TG-1801 represent diverse CD 47-targeting strategies discussed in this review (Table 1) [21,22,106]. For a broader overview, the work of Zi-Han Ye et al. provides a detailed analysis of current CD47-targeted approaches [107]. Magrolimab, a humanized anti-CD47 monoclonal antibody, was evaluated in combination with venetoclax plus either azacitidine or mitoxantrone/etoposide/cytarabine (MEC) in a Phase 2 trial involving patients with difficult-to-treat acute myeloid leukemia (AML) [22]. While the regimens demonstrated manageable safety profiles, efficacy was modest and comparable to standard therapies [22]. Higher rates of anemia, a known on-target effect of CD47 blockade, were observed but managed with supportive care. In relapsed/refractory AML, slight survival improvements likely resulted from increased rates stem cell transplantation [22]. Beyond inhibiting phagocytosis, CD47 influences antigen presentation, T cell activation, and tumor angiogenesis, highlighting its broader role in immune evasion. Bispecific strategies, such as TG-1801, aim to reduce red blood cell binding while preserving antitumor effects. Nevertheless, magrolimab’s development in AML was halted after similarly modest outcomes in the Phase 3 ENHANCE-3 trial, although CD47 remains under active investigation [22]. To improve safety and specificity, alternative CD47-targeting approaches have emerged. Second humanized monoclonal antibody, lemzoparlimab, binds a distinct CD47 epitope, reducing epitope, reducing cell affinity and anemia risk [21]. Early-phase studies in AML, myelodysplastic syndromes (MDS), non-Hodgkin lymphoma (NHL), and multiple myeloma have demonstrated favorable safety and preliminary efficacy, justifying continued development [21]. TG-1801, a bispecific antibody targeting both CD19 and CD47, was assessed in a first-in-human trial in patients with B-cell lymphoma, enabling selective engagement of malignant B cells while blocking immune evasion [106]. Despite progress in hematologic malignancies CD47-targeted therapies remain untested in AAA. Given CD47’s role in immune modulation, repurposing these strategies for AAA could offer novel therapeutic opportunities. Lemzoparlimab exemplifies epitope-specific engineering to reduce off-target effects while maintaining efficacy, while TG-1801 highlights the potential of bispecific formats for enhanced precision, a concept further explored in our therapeutic proposal.

## 8. CD47 as a Therapeutic Target in Cardiovascular Disease

CD47–TSP1 interactions are increasingly recognized as central mediators of cardiovascular and systemic diseases, reflecting a broader research trend driven by the success of CD47-targeted therapies in oncology [108,109,110,111]. CD47 regulates cardiovascular homeostasis by controlling cell adhesion, nitric oxide (NO) signaling, immune surveillance, and endothelial–leukocyte interactions crucial for vascular integrity [112,113,114]. In pathological states such as atherosclerosis, myocardial infarction, ischemia–reperfusion injury (IRI), pulmonary hypertension, and heart failure, CD47 is frequently upregulated, thereby impairing efferocytosis, sustaining inflammation, and limiting reparative angiogenesis. These processes contribute to plaque instability, tissue ischemia, fibrosis, and chronic vascular injury [114,115,116,117]. In atherosclerosis, CD47 overexpression on apoptotic cells inhibits SIRPα-dependent macrophage efferocytosis, leading to accumulation of necrotic debris and heightened inflammation, its inhibition reduces plaque progression and necrotic core size in murine models such as ApoE^−^/^−^ mice [118]. Similarly, in pulmonary arterial hypertension (PAH), antibody-mediated CD47 blockade lowers right ventricular systolic pressure and attenuates hypertrophy, effects attributed partly to suppression of TSP1 and restoration of caveolin-1 levels in pulmonary tissue [119]. In pressure-overload heart failure, loss or inhibition of CD47 improves both diastolic and systolic cardiac performance by downregulating the CaMKII (Ca^2+^/calmodulin-dependent protein kinase II)–HDAC3 (histone deacetylase 3) signaling pathway in cardiomyocytes, thereby reducing maladaptive cardiac remodeling [120]. These molecular changes translate into enhanced cardiac function and reduced maladaptive remodeling. In ischemic injury, CD47–TSP1 interaction disrupts the NO (nitric oxide)–cGMP (cyclic guanosine monophosphate) signaling pathway, impairing vascular relaxation and tissue reperfusion. CD47 knockout or blockade has been shown to enhance reperfusion and angiogenesis in ischemic models including hindlimb ischemia and skin flap transplantation in both mice and pigs [116,121]. Additionally, in inflammatory valvular diseases resembling rheumatic carditis, CD47 is upregulated on apoptotic cells, and blocking CD47 improves macrophage-mediated clearance, reduces pro-inflammatory cytokine production (e.g., IL-6, TNF-α), and limits fibrosis [120]. Age-related endothelial dysfunction, marked by increased CD47 and TSP1 expression, leads to impaired angiogenesis, diminished blood flow, and metabolic disturbances such as glucose intolerance. Blocking CD47 can restore vascular and metabolic function in aged mice [115].

Beyond cardiovascular pathology, CD47 contributes to cancer, autoimmune diseases, metabolic disorders, and infection [120,122]. In malignancies, CD47–SIRPα engagement provides a “don’t eat me” signal that inhibits phagocytosis of tumor cells. Therapeutic antibodies such as Hu5F9G4, CC90002, and SRF231 are in advanced clinical trials, demonstrating promising efficacy and manageable safety profiles [122]. In autoimmune conditions, including systemic lupus erythematosus (SLE), central nervous system autoimmunity, and autoimmune vasculitis, CD47 overexpression exacerbates inflammation, while blockade enhances efferocytosis and promotes immune resolution, thereby attenuating disease severity [123,124]. In metabolic diseases, CD47 fosters adipose tissue inflammation, hepatic steatosis, and kidney lipotoxicity, while inhibiting pancreatic β-cell insulin secretion, whereas inhibition improves metabolic homeostasis and glucose regulation in preclinical models [125]. Furthermore, CD47 blockade enhances innate and adaptive immune responses during infectious challenges, facilitating pathogen clearance without significant toxicity [120].

Collectively, current evidence positions CD47 as a dual-function molecule: a homeostatic regulator essential for maintaining vascular and immune system balance and a pathogenic driver when dysregulated across diverse diseases. TSP1 acts as a key ligand amplifying CD47-mediated effects, modulating processes such as inflammation, cellular clearance, and tissue remodeling [108,109,110,111]. Translational strategies targeting the CD47–TSP1 axis, particularly repurposing oncology-derived antibodies and gene-silencing techniques, hold considerable promise for treating cardiovascular and systemic inflammatory disorders. Early-phase clinical trials in cardiovascular disease are anticipated to clarify the safety, dosing, and therapeutic potential of these interventions [120,122].

In summary, CD47 has emerged as a pivotal regulator of immune signaling, vascular function, and cellular homeostasis across a wide range of pathological contexts. While its role in cancer immunotherapy is well established, expanding evidence underscores its involvement in cardiovascular disease progression, autoimmunity, metabolic dysfunction, and infection. The TSP1–CD47 axis remains central to these processes, influencing disease outcomes through modulation of efferocytosis, inflammation, vasodilation, and tissue repair. Continued clinical investigation is critical to translate these promising preclinical findings into safe and effective therapies for human disease.

In the specific context of AAA, combining such immune-targeted interventions with established clinical measures, screening, risk factor control, and evidence-based size thresholds could enable earlier, patient-tailored treatment before irreversible wall weakening occurs [24,126].

## 9. Future Applications: A Novel Treatment Proposal

**Figure 6:**
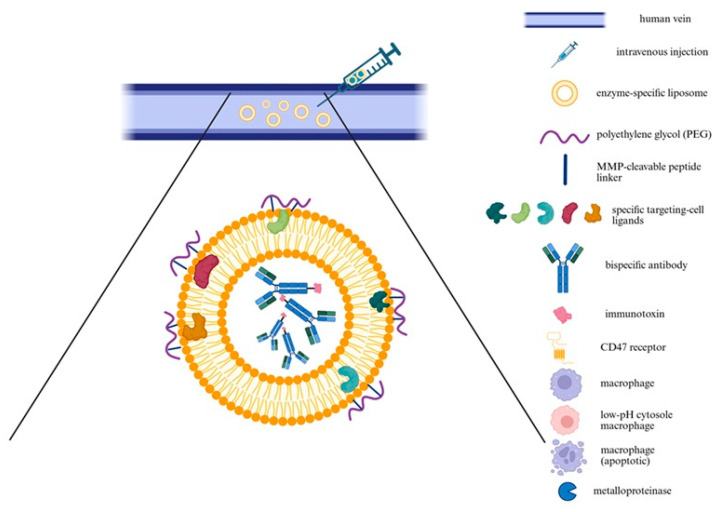
Schematic representation of a targeted immunoliposome delivery system. The diagram illustrates intravenous injection of MMP-responsive immunoliposomes designed for targeted delivery to macrophages in diseased vascular tissue, such as an aneurysmal aorta. Liposomes are coated with PEG linked via MMP-cleavable peptides, providing stealth properties during systemic circulation. At MMP-rich pathological site, enzymatic cleavage removes PEG, exposing targeting ligands and enhancing selective uptake by macrophages. The liposomal cargo includes bispecific antibodies and immunotoxins targeting CD47 and macrophage-specific markers, promoting apoptosis of pathogenic macrophages while minimizing off-target effects. Created in BioRender. Janas, K. (2025) https://BioRender.com/6vjogx7 (accessed on 30 October 2025).

**Figure 7:**
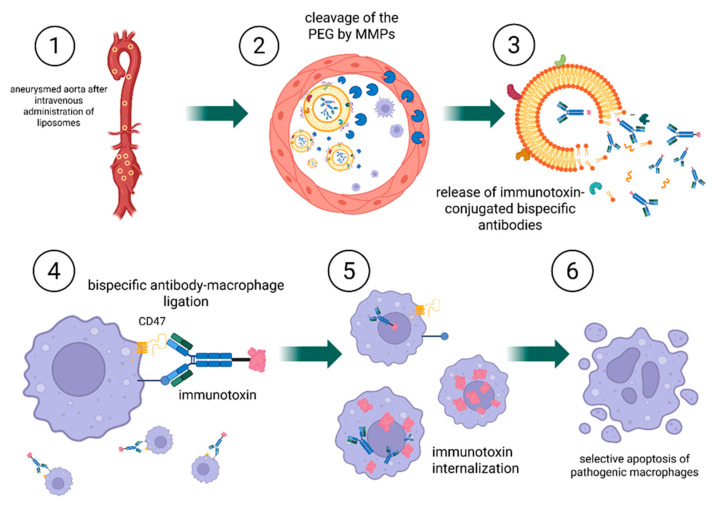
Mechanism of targeted CD47-directed immunoliposome delivery and macrophage elimination. 1. Following intravenous administration, PEGylated, MMP-responsive immunoliposomes circulate systemically and reach the AAA tissue, characterized by local overexpression of MMPs; 2. At the disease site, MMPs cleave the peptide linker, removing the PEG coating and exposing macrophage-targeting ligands; 3. Liposomal destabilization results in the release of immunotoxin-conjugated bispecific antibodies, which diffuse into the affected tissue; 4. Bispecific antibodies bind selectively to macrophage surface receptors, including CD47 and a macrophage-specific antigen; 5. Immunotoxins are internalized via endocytosis; 6. Lysosomal degradation releases the immunotoxin intracellularly, inducing selective apoptosis of pathogenic macrophages. Created in BioRender. Janas, K. (2025) https://BioRender.com/pvm1g30 (accessed on 2 November 2025).

**Figure 8:**
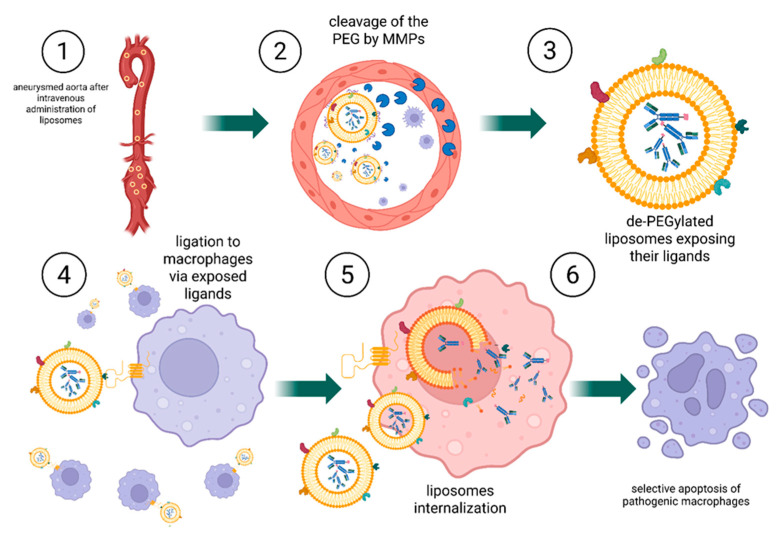
Mechanism of targeted CD47-directed immunoliposome delivery and macrophage elimination. 1. Following intravenous administration, PEGylated, MMP-responsive immunoliposomes circulate through the bloodstream including AAA tissue, where MMPs are locally overexpressed; 2. At the disease site, MMPs cleave the peptide linker, resulting in PEG shedding and exposure macrophage-targeting ligands on the liposomal surface; 3. A crucial distinction occurs at this stage: liposomes with exposed ligands are now capable of binding to nearby macrophages; 4. Immunoliposomes bind to CD47 receptors on the macrophage surface via the exposed targeting ligands; 5. The bound liposomes are internalized through endocytosis and subsequently undergo intracellular disassembly; 6. Lysosomal degradation of the liposomes leads to the release of the immunotoxin, inducing selective apoptosis of macrophages. Created in BioRender. Janas, K. (2025) https://BioRender.com/bsxk4dn (accessed on 3 November 2025).

As a conceptual proposition, we outline two potential strategies for targeted drug delivery (shown in [Figure 6, Figure 7 and Figure 8]) that could combine bispecific targeting of CD47 with a macrophage-specific marker and the use of liposome-encapsulated immunotoxins to improve selectivity and therapeutic efficacy.

### 9.1. Targeted Delivery Approach

Both therapeutic strategies utilize enzyme-specific liposomes that accumulate in the aneurysmal aortic wall. Liposomes, first described by Bangham in 1961, gained prominence as drug carriers in the 1980s due to improved bioavailability and reduced systemic toxicity [98]. Early preclinical studies confirmed the feasibility of encapsulating chemotherapeutic, prompting efforts to enhance vesicle stability and loading capacity [98]. A major milestone was the FDA approval of Doxil in 1995, the first liposomal formulation for clinical use, establishing liposomes as a viable platform therapying oncology, particularly for ovarian and breast cancers [98]. More recently, in 2024, the FDA approved NALIRIFOX —a multi-agent regimen including liposomal irinotecan (Onivyde), for first-line treatment of metastatic pancreatic adenocarcinoma [127]. This marked a significant advance in systemic therapy for this aggressive malignancy, highlighting continued innovation in liposomal drug delivery [127]. In both approaches, liposomes are administered intravenously and engineered to respond to MMPs, which are overexpressed in pathological tissues such as the AAA. A protective polyethylene glycol (PEG) coating, attached via an MMP-cleavable peptide linker, confers stealth properties during circulation by reducing nonspecific uptake. At the target site, MMP-mediated cleavage removes PEG, exposing ligands or fusogenic peptides that promote cellular uptake, particularly by macrophages [128,129]. This concept was demonstrated using MMP-sensitive liposomes containing a polyethylene glycol (PEG)-shielded TAT (Trans-Activator of Transcription) peptide. Upon enzymatic cleavage by MMPs, the TAT peptide was unmasked, significantly enhancing cellular internalization [129].

Anti-CD47 therapy involves monoclonal antibodies, which may cause systemic toxicity [130]. Therefore, precise delivery is critical to reduce adverse effects and improve efficacy. Although antibody-mediated targeting improves liposomal specificity, it does not substantially affect intracellular trafficking pathways, both targeted and untargeted liposomes are generally internalized via endocytosis and degraded in lysosomes [131]. Localized delivery, e.g., MMP-activated liposomes targeted the aneurysmal wall, could minimize systemic exposure and facilitate the use of immunotherapies in non-oncological contexts.

To evaluate the potential of liposomal delivery for CD47-targeted therapy, we analyzed current studies. Notably, Imiquimod-loaded liposomes incorporating Fc-CV1 and targeting CD47 demonstrated sustained release, selective tumor accumulation, and potent efficacy in a colon cancer model [98]. This effect was mediated by innate immunity activation and blockade of the CD47 targeting with liposomal delivery and suggests potential synergy with immune checkpoint inhibitors [132].

Immunoliposomes offer improved initial targeting by resisting protein corona formation and binding to target cells, as shown in cervical cancer model [131]. However, this specificity decreases over time due to ligand dissociation, epitope masking by plasma proteins, and dynamic corona remodeling, which alters nanoparticle identity [131]. Consequently, nonspecific uptake mechanisms like as macropinocytosis become dominate [131]. Macropinocytosis, a bulk endocytosis process, is constitutively active in cancer cells with oncogenic RAS mutations, supporting protein uptake for tumor growth [133]. It also promotes immune evasion by activating mTOR (mechanistic target of rapamycin), increasing PD-L1 (programmed death-ligand 1) expression, and inhibiting T-cell activation. Therefore, targeting macropinocytosis may enhance therapy by both inhibiting tumor metabolism and restoring immune responses [133]. At the same time, its role in enhancing nanoparticle uptake makes it a double-edged sword-supporting early delivery while also benefiting cancer survival. Thus, optimizing timing and specificity of delivery systems remains key to effective treatment [131].

Strategy 1:

In the first strategy, the liposome releases an immunotoxin after activation by MMPs in the aneurysm environment. The immunotoxin consists of bispecific antibodies: one arm targets the CD47 receptor, while the other binds to a macrophage-specific surface marker. Following released, the immunotoxin diffuses through the tissue and selectively binds to macrophages, inducing their targeted elimination. In this approach, the immunotoxin mediates cell-specific targeting, whereas the liposome functions as an enzyme-responsive carrier enabling localized and controlled release.

Strategy 2:

The second strategy differs fundamentally in its mechanism. In this case, the entire liposome is engineered to recognize and bind to macrophages following activation by MMPs. Upon removal of the protective PEG layer, exposed ligands on the liposomal surface direct binding to macrophages. The liposome is subsequently internalized by the cell. Within the acidic intracellular environment of the macrophage, the pH-sensitive liposomal membrane becomes destabilized, leading to the release of the immunotoxin and, ultimately, cell death. This dual-trigger design, combining MMP sensitivity for extracellular activation with pH sensitivity for intracellular release, enables highly selective delivery of therapeutic agents to target cells.

The principal distinction between these two approaches lies in their targeting mechanism: in the first, the released immunotoxin independently locates and affects macrophages, whereas in the second, the liposome itself is responsible for targeting, internalization, and delivery of the therapeutic payload.

To summarize and compare the strategies discussed above, we provide the following clarification. In the first design, the cleavable PEG moieties are anchored in such a way that enzymatic removal (e.g., by MMPs) directly destabilizes the lipid bilayer, resulting in the extracellular rupture of the liposome and ensuing release of antibody-toxin payloads into the tumor microenvironment. In contrast, the second design decouples PEG removal from membrane disruption: PEG cleavage merely unmasks targeting ligands on a still-intact liposome, enabling ligand-mediated binding and cellular uptake (e.g., by macrophages). Only after endocytosis into acidic endosomal/lysosomal compartments does the liposomal membrane destabilize (via pH-sensitive lipids), releasing the therapeutic payload intracellularly. This dichotomy between immediate extracellular release and delayed intracellular release is rooted in the distinct roles of PEG. In the first case, PEG functions as a stabilizer whose removal induces membrane rupture, whereas in the second case, PEG acts as a “stealth mask” that exposes binding ligands while preserving membrane integrity until internalization.

### 9.2. Safety Considerations and Translational Challenges of CD47 Blockade in AAA

While CD47-targeted therapies have demonstrated clinical feasibility in oncology, their translation into vascular medicine raises important safety considerations. In cancer trials, anemia is the most frequently reported adverse event, resulting from antibody-mediated opsonization and macrophage clearance of red blood cells, a mechanism consistent with the “on-target” pharmacodynamic effect of CD47 blockade [134,135,136]. Although generally reversible and manageable with dose titration or transfusion support, this effect poses a particular challenge in elderly AAA patients, who often present with limited hematopoietic reserve, chronic inflammation, and coexisting atherosclerotic or ischemic disease.

To mitigate these risks, next-generation CD47-targeted agents employ epitope-specific antibodies (e.g., lemzoparlimab) with reduced red blood cell affinity, or bispecific constructs that restrict binding to selected immune or vascular targets [137]. Localized or enzyme-responsive delivery systems, such as the MMP-activated liposomes proposed herein, may further limit systemic exposure and hematologic toxicity by confining drug activity to diseased vascular segments. Nevertheless, preclinical validation in aged and comorbid animal models remains essential to establish safety and pharmacokinetic behavior in a population that closely mirrors typical AAA patients. Addressing these issues is critical to bridge the translational gap between promising molecular design and clinical applicability.

### 9.3. Translational Limitations of Liposomal Delivery in AAA

Although liposomal formulations such as Doxil and liposomal irinotecan (Onivyde/NALIRIFOX regimen) have achieved regulatory approval in oncology, their successful translation cannot be directly extrapolated to vascular diseases such as AAA. The aneurysmal aortic wall presents a unique biological and biomechanical environment characterized by altered hemodynamics, high wall shear stress, degraded extracellular matrix, and the frequent presence of intraluminal thrombus [138,139]. These factors substantially limit nanoparticle penetration beyond the luminal surface and may prevent effective delivery into the adventitia, where macrophage-driven inflammation predominates [140]. Moreover, the risk of uncontrolled necrosis or hemorrhage secondary to off-target cytotoxicity is particularly concerning in a structurally weakened vessel wall.

To address these translational barriers, future work should focus on hemodynamics-informed carrier design (e.g., shear-sensitive or thrombosis-adaptive nanoparticles), local intra-adventitial administration during endovascular procedures, and controlled-release systems responsive to matrix metalloproteinase activity. Acknowledging these limitations is essential for a realistic appraisal of the current technological readiness level of macrophage-targeted immunoliposomes in AAA therapy.

## 10. Immunotoxins

A key distinction of our proposed strategy is that it does not aim to stimulate an immune response. In AAA, where inflammation drives pathology, further immune activation is undesirable. Instead of using monoclonal antibody therapies to trigger immune effector functions, our approach targets the selective elimination or suppression of disease-associated macrophage to minimize the inflammatory burden.

Immunotoxins are chimeric fusion proteins comprising a targeting domain, typically an antibody or fragment binding selectively to a cell-surface antigen, and a cytotoxic domain that induces cell death upon internalization [141,142]. After endocytosis, immunotoxins are degraded in lysosomes, releasing the active toxin into the cytosol [143]. In this study, the authors showed that the GPC3-targeting immunotoxin HN3-mPE24 induced potent cytotoxicity in GPC3-positive hepatocellular carcinoma (HCC) cells, confirming its therapeutic potential. The selective overexpression and rapid endocytosis of GPC3 in HCC make it an ideal target for antibody-based therapeutics. These findings support the GPC3-targeted immunotoxins in HCC treatment, particularly in tumors resistant to conventional therapies and suggest that this approach may be adaptable for other diseases requiring targeted cell elimination [143].

### 10.1. Macrophage Specific Surface Markers

This section summarizes macrophage-specific surface markers reported in AAA tis-sue that may hold potential as therapeutic targets. Several of these markers, including triggering receptor expressed on myeloid cells 2 (TREM2) and CD86, have been explored in immunomodulatory strategies and may hold translational potential for targeted intervention.

### 10.2. Presence of TREM2^+^ and CD86^+^ Macrophage Subsets in AAA Models

TREM2 is predominantly expressed by macrophages, where it plays essential roles in immune regulation and cellular homeostasis [144]. Single-cell transcriptomic analyses have identified TREM2^+^ macrophages in both murine and human AAA models [144] conserved across species and acting as a key mediators of arterial wall degeneration [144]. Sunita Keshari et al. demonstrated that adding TREM2 blockade to neoantigen cancer vaccines or immune checkpoint therapies enhanced tumor-specific CD8^+^ T-cell expansion and improved tumor control [145]. In murine melanoma models, intertumoral CX3CR1^+^ CD206^+^ macrophages expressing TREM2 suppressed vaccine efficacy, a suppression reversed by TREM2-blocking antibodies [145]. CD86^+^ macrophages, akin to TREM2^+^ subsets, have also been detected in AAA models, reinforcing their potential as therapeutic targets and supporting macrophage-directed strategies in AAA treatment [144,146]. While bispecific antibodies are established in oncology and autoimmune disease, their application in vascular disorders such as AAA remains limited. Given current insights into AAA pathophysiology, bispecific approaches may align well with disease mechanisms, providing a plausible framework for future therapeutic development.

### 10.3. Challenges and Adjuvant Treatment

Given the potential for ongoing recruitment of inflammatory cells within the aortic microenvironment, even after depletion of existing macrophage, repeated dosing may be required to sustain therapeutic efficacy. To enhance outcomes, we propose incorporating adjuvant strategies, such as M2-polarized macrophages administration and statin therapy [94]. Although complete elimination of pro-inflammatory M1 macrophages in unlikely, restoring the disrupted M1/M2 ratio, previously discussed as a hallmark of AAA pathology, remains a key objective [147]. Combining targeted M1 depletion with M2 supplementation may foster a stable, anti-inflammatory environment and improve treatment durability. However, the potential risk that aggressive macrophage depletion could further weaken an already thinned aneurysmal wall and promote rupture should not be underestimated. For this reason, the proposed therapy is intended to be applied at early disease stages, when structural integrity is maintained and adverse effects are less likely.

Statins, with their pleiotropic anti-inflammatory effects and ability to suppress MMP expression, are promising adjuvants [148]. Similarly, angiotensin-converting enzyme (ACE) inhibitors and angiotensin II receptor blockers (ARBs), widely used in cardiovascular care, have been proposed as supportive therapies [70]. It is also worth noting that antibiotic therapy has been investigated for its potential to slow aneurysm expansion by limiting elastin degradation and local inflammation [70]. Based on current evidence, statins, ACE inhibitors, and ARBs continue to represent integral components of medical management in patients with AAA [149,150].

## 11. Future Directions

Abdominal aortic aneurysm remains a major unmet clinical challenge due to the lack of effective pharmacological therapies capable of halting disease progression. Emerging evidence identifies the CD47–TSP1 axis as a pivotal upstream regulator of chronic inflammation, defective efferocytosis, and extracellular matrix degradation—processes central to aneurysm pathogenesis. By modulating macrophage activation and sustaining inflammatory loops, this pathway represents a promising therapeutic target addressing the immunological and molecular drivers of AAA rather than merely its structural manifestations. Preclinical studies, together with evidence from vascular and oncologic contexts, support the translational potential of CD47 blockade using monoclonal antibodies such as magrolimab or lemzoparlimab, while TSP1 may serve as both an effector molecule and a biomarker of disease activity.

However, significant knowledge gaps persist. The precise molecular mechanisms linking CD47–TSP1 signaling to macrophage polarization and extracellular matrix remodeling require validation in human tissues and large-animal models. Furthermore, the temporal dynamics of CD47 expression during aneurysm development remain undefined, as do the pharmacokinetics and vascular safety of anti-CD47 agents in chronic, non-malignant inflammatory settings. Future research should therefore prioritize longitudinal analyses of CD47/TSP1 expression in clinical specimens, targeted delivery strategies to mitigate systemic toxicity, and the development of macrophage-restricted or bispecific antibodies to enhance selectivity.

Integration of CD47/TSP1 modulation with molecular imaging and immune phenotyping may further accelerate translation by enabling noninvasive monitoring of disease activity and therapeutic response. Combining CD47-targeted interventions with approaches that modulate Treg function or macrophage polarization could also yield synergistic immunomodulatory effects, advancing a more holistic immune-centered therapeutic paradigm.

Future research should additionally focus on refining the design, selectivity, and translational applicability of CD47-targeted immunotoxin strategies for AAA. Preclinical studies are needed to confirm the efficacy and safety of macrophage-selective immunotoxins in large-animal models that better replicate the hemodynamic and inflammatory microenvironment of human AAA. Particular attention should be given to optimizing toxin potency to achieve macrophage depletion without affecting structural vascular cells such as smooth muscle or endothelial cells.

The pharmacokinetics, biodistribution, and immunogenicity of enzyme-responsive liposomal carriers also require systematic evaluation under chronic inflammatory conditions. Advances in molecular imaging—such as positron emission tomography (PET) or near-infrared tracers—could be leveraged to visualize macrophage-targeted delivery and monitor treatment response in vivo. Moreover, integrating CD47-directed immunotoxins with complementary modalities—including anti-inflammatory agents, M2 macrophage transfer, or statins—may enhance vascular repair and immune resolution.

Finally, precision-medicine approaches incorporating genetic and transcriptomic profiling of AAA tissue could identify patient subsets most likely to benefit from macrophage-targeted therapies. Collectively, these directions will be critical for bridging the gap between mechanistic insight and clinical translation of immunotoxin-based and CD47-directed interventions in vascular disease. Ultimately, early-phase clinical trials assessing feasibility, safety, and biomarker responses to CD47-targeted therapies in patients with small AAAs will be essential to validate their disease-modifying potential and to transform the management of this currently untreatable condition.

## 12. Discussion

The TSP1–CD47 signaling axis is increasingly recognized as a driver of AAA pathogenesis, integrating macrophage-driven inflammation, ECM degradation, and immune dysregulation. While classical models emphasize the role of MMPs, particularly MMP-2 and MMP-9, recent evidence indicates that the upstream immune checkpoint CD47 and its ligand thrombospondin-1 regulate multiple downstream pathways that collectively promote vascular wall weakening [20,40,41,94]. Persistent activation of this axis sustains macrophage polarization toward the M1 phenotype, amplifies cytokine release, and perpetuates the self-reinforcing inflammatory loop that underlies aneurysm formation [18,20,36]. Consequently, modulation of the CD47–TSP1 interaction represents a novel and promising therapeutic paradigm that targets the immune drivers of disease progression rather than its structural consequences.

In the broader therapeutic landscape, several experimental strategies have been explored to limit AAA growth and rupture risk. Anti-cytokine therapies targeting TNF-α, IL-1β, or IL-6 have shown partial success in animal models by reducing inflammation and ECM degradation [96,97,98]. For instance, IL-1β inhibition with anakinra or canakinumab attenuated aortic dilation in murine models, whereas IL-6 receptor blockade (tocilizumab) reduced inflammatory cell infiltration and MMP activity [97,98]. However, clinical translational has been hampered by systemic immunosuppression, short-lived effects, and inconsistent outcomes in human studies [99]. Similarly, MMP inhibitors such as doxycycline, marimastat, and batimastat, initially showed promise but failed in large clinical trials due to toxicity and pathway redundancy, underscoring the limitations of single-enzyme targeting approaches [70,72,79].

Cell-based therapies have also gained attention, particularly the use of MSCs and M2-polarized macrophage transfer, both aimed at restoring immune balance and promoting vascular repair through paracrine signaling [94]. Preclinical models suggest that M2 macrophages reduce IL-1β and IL-6 levels, limit elastin degradation, and downregulate MMP-9, thereby stabilizing the aortic wall [94]. Likewise, MSC administration has been shown to improve endothelial regeneration and mitigate inflammation in aneurysmal models [147,148]. Despite encouraging results, these approaches face significant challenges related to delivery efficiency, cell survival, and phenotypic stability within the inflammatory aneurysmal environment [151].

To reinforce the relevance of immune-targeted interventions, recent findings on immune cell subsets and potential therapeutic targets in AAA merit consideration. A 2023 study demonstrated that CD8^+^ T cells, regulatory T cells (Tregs), and macrophages are critically involved in AAA pathogenesis. While Tregs appear to limit aneurysm progression, CD8^+^ T cells exert pro-inflammatory effects, making both populations attractive immunomodulatory targets for therapeutic exploration [152]. Furthermore, a 2024 study revealed that CD8^+^ T-cell–derived IFN-γ promotes AAA formation in mice, suggesting that regulation of this pathway could represent a novel therapeutic direction [153]. Modulation of CD8^+^ T-cell activity, in parallel with targeting the CD47–TSP1 axis, may therefore offer complementary avenues for mitigating vascular inflammation and ECM remodeling. In addition, recent preclinical work identified Tregs as promising immunotherapeutic targets: a 2025 study using CAR-Treg cells directed against VCAM-1 demonstrated attenuation of aneurysm progression, indicating that modulation of Treg number or function may help limit disease development [154]. Although these findings remain preliminary, simultaneous targeting of Treg pathways and the CD47–TSP1 axis represents a rational, immune-based strategy to restore vascular immune homeostasis in AAA.

Compared with these modalities, CD47–TSP1 targeting offers a distinct and integrative mechanism acting upstream of both cytokine signaling and proteolytic activation. By restoring macrophage efferocytosis, normalizing nitric oxide signaling, and dampening chronic inflammation, CD47 blockade has the potential to interrupt multiple convergent pathways driving AAA pathogenesis [18,20,40,41]. This approach differs fundamentally from conventional anti-inflammatory strategies, which often suppress systemic immunity, by instead re-establishing physiological immune homeostasis within the vascular wall. Furthermore, the translational feasibility of CD47-targeted agents such as magrolimab and lemzoparlimab, already evaluated in oncology, provides a strong foundation for repurposing these therapies in vascular disease with established safety profiles and pharmacodynamics [21,22,106].

Advances in molecular imaging have created new opportunities for monitoring disease evolution and evaluating the efficacy of targeted interventions. In a 2024 study, rats with AAA were imaged using positron emission tomography–computed tomography (PET-CT) with ^18F-FDG and ^18F-NaF [155]. The uptake of ^18F-NaF and ^18F-FDG, as well as the calcification volume, correlated with AAA development, with ^18F-NaF (^18F-sodium fluoride) uptake emerging the strongest predictor [155]. These findings underscore the potential of molecular imaging to identify suitable candidates for emerging immunotherapies and to enable the longitudinal assessment of treatment response.

Moreover, recent genome-wide association meta-analysis identified 121 independent AAA risk loci, highlighted potential therapeutic targets such as proprotein convertase, subtilisin/kexin-type 9 (PCSK9) [156]. Integrating genetic signatures with immune phenotyping could facilitate patient stratification and support the rationale for CD47- and macrophage-targeted liposomal immunotherapies in selected AAA populations [156]. Such an integrative, precision-medicine approach could refine therapeutic selection and maximize benefit while minimizing adverse effects.

Finally, integration of CD47-directed therapy with other emerging modalities could further enhance efficacy. Combining CD47 blockade with M2 macrophage therapy, statins, or ACE inhibitors may provide synergistic benefits by coupling immune modulation with vascular protection [70,148]. Likewise, delivery via matrix metalloproteinase-activated or macrophage-targeted liposomes could maximize local drug concentration while minimizing systemic toxicity [128,129]. When viewed alongside existing therapeutic concepts, CD47–TSP1 modulation emerges as a next-generation, immune-centered strategy that aligns with the multifactorial nature of AAA and may ultimately redefine its pharmacologic management.

## 13. Conclusions

Abdominal aortic aneurysm remains a major unmet clinical challenge due to the lack of effective pharmacological therapies. The CD47–TSP1 signaling axis functions as a pivotal upstream regulator of inflammation, defective efferocytosis, and ECM degradation, key processes in AAA pathogenesis. By modulating macrophage activation and sustaining chronic inflammatory loops, this pathway represents a promising therapeutic target that addresses the immunological and molecular drivers of AAA rather than its structural manifestations.

Preclinical and translational studies support the feasibility of targeting this axis using anti-CD47 monoclonal antibodies such as magrolimab or lemzoparlimab, with TSP1 serving as both an effector molecule and a potential biomarker. Advances in molecular imaging, including ^18F-NaF PET-CT, may facilitate the identification of suitable candidates for immunotherapies and enable longitudinal monitoring of treatment response. Furthermore, the integration of genetic and immune profiling, highlighting novel targets such as PCSK9, could support patient stratification and guide precision-based vascular immunotherapy.

Future research should prioritize the validating of predictive biomarkers, characterization of the temporal dynamics of CD47–TSP1 signaling in human AAA, and evaluation long-term vascular safety in large-animal and early-phase clinical studies. Collectively, targeting the CD47–TSP1 axis represents a new disease-modifying therapeutic direction for AAA that could complement or delay surgical intervention and ultimately lay the foundation for the first effective pharmacological approach in AAA management.

## Figures and Tables

**Table 1:** Summary of CD47-targeted antibodies in cancer therapy.

Drug Name	Type	Therapeutic Application/Clinical Use	Mechanism of Action
Magrolimab	Humanized monoclonal antibody (IgG4) targeting CD47	Acute Myeloid Leukemia (AML)–evaluated in combination with venetoclax + azacitidine or MEC in Phase 2 and 3 trials	On-target anemia observed; development in AML discontinued after modest Phase 3 results
Lemzoparlimab	Humanized monoclonal antibody (IgG4) targeting CD47	Binds a distinct CD47 epitope compared to magrolimab, reducing red blood cell binding and anemia risk	Demonstrates favorable safety and preliminary efficacy; under continued investigation
TG-1801	Bispecific antibody targeting CD19 and CD47	Simultaneously targets CD19 and blocks CD47, enabling selective engagement of malignant B cells	Enhances precision via dual-antigen targeting; a promising strategy for improved selectivity

## Data Availability

No new data were created or analyzed in this study. Data sharing is not applicable to this article.

## Abbreviations

The following abbreviations are used in this manuscript:

AAA	Abdominal aortic aneurysm
ACE	Angiotensin-converting enzyme
ACS	Acute coronary syndrome
ADAMTS1	A disintegrin and metalloproteinase with thrombospondin motifs 1
AML	Acute myeloid leukemia
ARB	Angiotensin II receptor blockers
CaMKII	Ca^2+^/calmodulin-dependent protein kinase II
CCL1	C-C motif ligand 2
CCR2	C-C chemokine receptor type 2
CD47	Cluster of Differentiation 47
CD86	Cluster of Differentiation 86
CKD	Chronic kidney disease
COMP	Cartilage oligomeric matrix protein
cGAS–STING	Cyclic GMP-AMP synthase–stimulator of interferon genes
cGMP	Cyclic guanosine monophosphate
CTD	Intracellular domain
CVD	Cardiovascular disease
DAMP	Damage-associated molecular patterns
ECM	Extracellular matrix
ECD	Extracellular domain
EGF-like repeat	Epidermal Growth Factor-like domain
HDAC3	Histone deacetylase 3
HCC	Hepatocellular carcinoma
IFNICs	Interferon-inducible cells
IgV	Immunoglobulin Variable domain
IAP	Integrin-associated protein
Ifnar1	Interferon alpha/beta receptor 1
IL	Interleukin
IL-1β	Interleukin-1 beta
IRI	Ischemia–reperfusion injury
iNOS	Inducible Nitric Oxide Synthase
ITIM	Immunoreceptor Tyrosine-based Inhibitory Motif
JAK–STAT	Janus kinase–signal transducer and activator of transcription
MDS	Myelodysplastic syndromes
MEC	Mitoxantrone, Etoposide, Cytarabine
MCP-1	Monocyte chemoattractant protein-1
MMP	Matrix metalloproteinases
MSS	Musculoskeletal syndrome
mTOR	Mechanistic Target of Rapamycin
NLC	Nanostructured lipid carrier
NF-κB	Nuclear Factor kappa-light-chain-enhancer of activated B cells
NHL	Non-Hodgkin lymphoma
NO	Nitric oxide
PAH	Pulmonary arterial hypertension
PD-L1	Programmed Death-Ligand 1
PEG	Polyethylene Glycol
PC	Procollagen homology domain
R-spondins	Roof plate–specific spondins
SCO	Subcommissural organ spondin
SH2	Src Homology 2 domain
SHP1	Src Homology region 2 domain-containing Phosphatase-1
SHP2	Src Homology region 2 domain-containing Phosphatase-2
SIRPα	Signal regulatory protein
SLE	Systemic lupus erythematosus
SS	Systemic sclerosis
TAT	Trans-Activator of Transcription
TIMP	Tissue inhibitors of metalloproteinases
TGF-β	Transforming growth factor
TMD	Transmembrane domain
TNF-α	Tumor necrosis factor-alpha
TREM	Triggering Receptor Expressed on Myeloid Cells 2
TSR	Thrombospondin Type 1 Repeats
TSP1	Thrombospondin-1
VEGF	Vascular Endothelial Growth Factor
VSMC	Vascular smooth muscle cell
